# Successful Limb Salvage in a Newly Diagnosed Diabetic Patient With Critical Limb Ischemia Managed With Percutaneous Transluminal Angioplasty and Stenting

**DOI:** 10.7759/cureus.104604

**Published:** 2026-03-03

**Authors:** Jaison Paul, Deebanshu Gupta, Manveer Singh

**Affiliations:** 1 Internal Medicine, Sharma Hospital, Hoshiarpur, IND; 2 Cardiology, Sarvodya Hospital, Jalandhar, IND; 3 Community Medicine, Sudha Medical College and Hospital, Kota, IND

**Keywords:** critical limb ischemia, diabetic foot ulcer, endovascular revascularization, percutaneous transluminal angioplasty, peripheral arterial disease, superficial femoral artery stenting, type 2 diabetes

## Abstract

Diabetic foot ulcers are a major cause of morbidity and limb loss, particularly when complicated by underlying vascular insufficiency. We report the case of a middle-aged male patient who presented with generalized weakness, epigastric discomfort, fever, and a chronic non-healing ulcer over the right foot. Initial evaluation revealed newly diagnosed type 2 diabetes mellitus, acute kidney injury secondary to dehydration, and a chronic diabetic foot ulcer. Despite stabilization with medical management, the ulcer failed to heal, prompting further vascular evaluation. Peripheral angiography demonstrated peripheral arterial occlusive disease (PAOD) with critical limb ischemia (CLI) involving the right lower limb. The patient subsequently underwent percutaneous transluminal angioplasty with stenting of the right superficial femoral artery, resulting in restoration of limb perfusion. Following revascularization, the ulcer showed progressive healing with significant clinical improvement. At follow-up, the patient had regained full functional mobility and resumed normal occupational activities. This case underscores the importance of early recognition of vascular insufficiency in diabetic foot ulcers and highlights the role of timely endovascular intervention in achieving limb salvage.

## Introduction

Diabetic foot ulcers represent one of the most serious complications of diabetes mellitus, affecting up to 25% of diabetic patients during their lifetime. These ulcers significantly contribute to morbidity, prolonged hospitalizations, and lower-limb amputations. The presence of peripheral arterial occlusive disease (PAOD) further compromises tissue perfusion, delays wound healing, and increases the risk of critical limb ischemia (CLI), a severe form of peripheral arterial disease characterized clinically by ischemic rest pain, non-healing ulcers, or gangrene attributable to objectively proven arterial insufficiency [1].

Diabetes mellitus often remains undiagnosed until complications arise, as hyperglycemia can remain asymptomatic for prolonged periods. Chronic non-healing foot ulcers may be the first clinical manifestation, particularly when complicated by underlying vascular disease. Up to one-third (≈19%-34%) of individuals with diabetes develop a foot ulcer in their lifetime, with peripheral arterial disease (ischemia) accounting for up to ~50% of diabetic foot ulcers and making them a leading cause of non-traumatic lower-limb amputations worldwide. Timely diagnosis of PAOD and prompt revascularization are essential to restore blood flow, promote wound healing, and prevent amputation [2-3].

We report a case of a newly diagnosed diabetic patient presenting with a chronic non-healing foot ulcer and CLI, successfully managed with endovascular revascularization using percutaneous transluminal angioplasty and stenting, resulting in complete limb salvage and ulcer healing without the need for surgical reconstruction or skin grafting.

## Case presentation

History

A 39-year-old male patient was admitted to Sharma Hospital, Garhdiwala, Punjab, India, on April 29, 2025, with complaints of restlessness and epigastric discomfort for three to four days, generalized weakness with reduced oral intake for a similar duration, fever for one day, and a non-healing ulcer over the right foot for four months. He had no previously documented history of diabetes mellitus, hypertension, coronary artery disease, or peripheral vascular disease. There was no prior history of chronic kidney disease. The patient was not on any long-term medications.

Clinical examination

**Figure 1:**
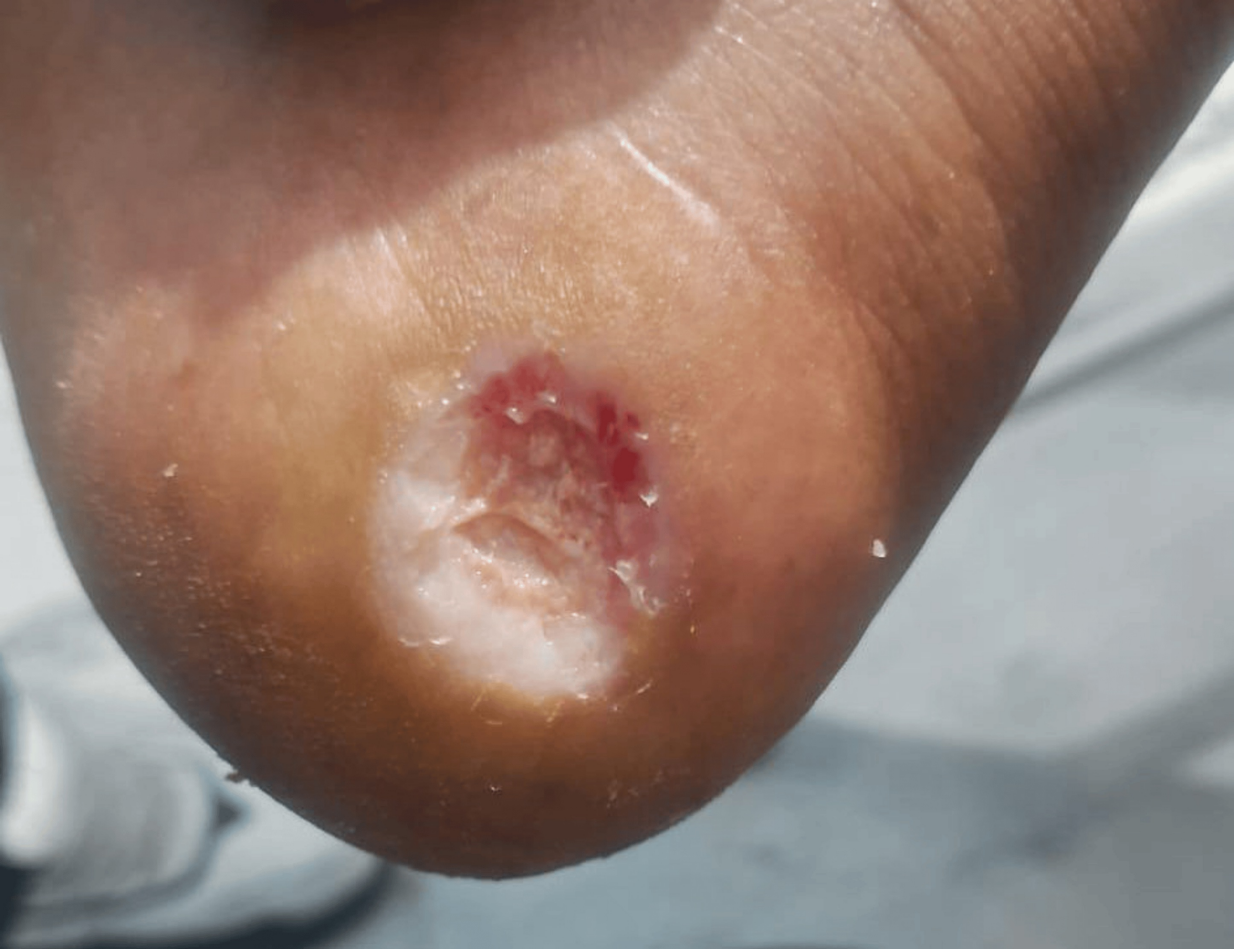
Baseline ischemic heel ulcer with slough and poor granulation

On admission, the patient was conscious but lethargic. Vital parameters revealed a blood pressure of 90/50 mmHg and a pulse rate of 78 beats per minute. Cardiovascular examination revealed normal heart sounds, and respiratory and neurological examinations were unremarkable. Local examination of the right foot showed a chronic ulcer with poor granulation tissue, as shown in Figure 1. Peripheral pulses in the right lower limb were diminished, raising suspicion of underlying vascular insufficiency.

Laboratory investigations

The patient's initial laboratory investigations are shown in Table 1.

**Table 1:** Laboratory investigations with reference ranges Hb: hemoglobin; TLC: total leukocyte count; PLT: platelet count; BU: blood urea; SCr: serum creatinine; SGOT (AST): serum glutamic-oxaloacetic transaminase (aspartate aminotransferase); SGPT (ALT): serum glutamic-pyruvic transaminase (alanine aminotransferase)); hpf: high power field; VDRL: Venereal Disease Research Laboratory test; RBS: random blood sugar

Parameter	Observed Value	Reference Range
Hemoglobin	13.2 g/dL	12–16 g/dL (female)
Total leukocyte count	7,600 /µL	4,000–11,000 /µL
Platelet count	123 × 10⁹/L	150–450 × 10⁹/L
Blood urea	70.42 mg/dL	15–40 mg/dL
Serum creatinine	1.81 mg/dL	0.6–1.2 mg/dL
SGOT (AST)	32.09 IU/L	5–40 IU/L
SGPT (ALT)	44.51 IU/L	7–56 IU/L
Urine examination	Pus cells 5–6 /hpf	0–5 /hpf
VDRL	Positive	Negative
Random blood glucose	211	<140 mg/dL

Based on clinical and laboratory findings, the patient was diagnosed with newly detected type 2 diabetes mellitus, acute kidney injury secondary to hypovolemia, and a chronic non-healing diabetic foot ulcer.

Initial management

The patient was managed with intravenous fluids for dehydration, broad-spectrum intravenous antibiotics, proton pump inhibitors, antiemetics, and supportive care. Renal parameters improved with hydration, and the patient’s general condition stabilized. However, the foot ulcer continued to show poor healing despite optimal medical management.

Further evaluation and intervention

**Figure 2:**
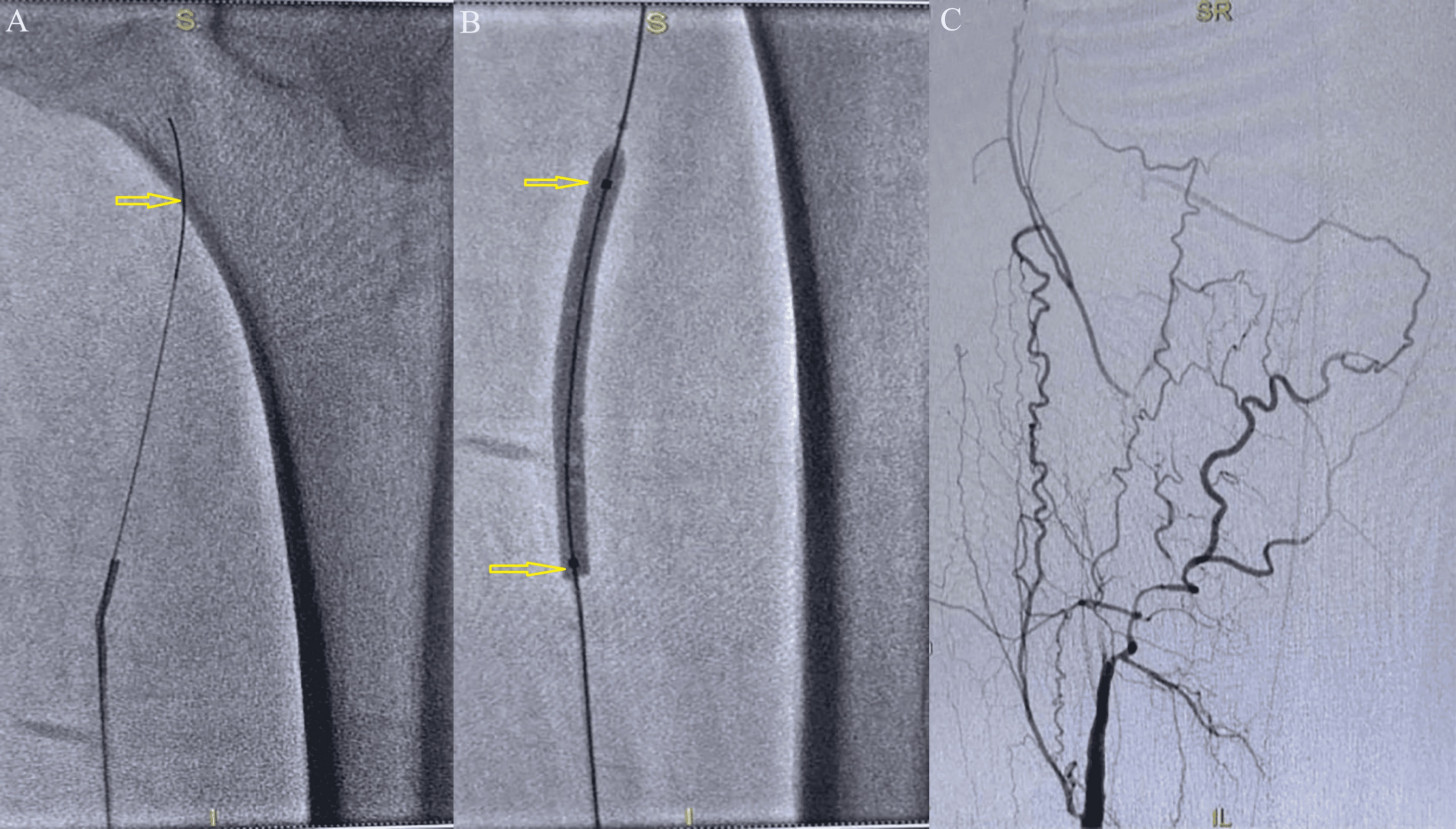
Angiography images A: Diagnostic angiography showing superficial femoral artery disease with poor distal runoff; B: Percutaneous transluminal angioplasty with a stent positioned in the right superficial femoral artery; C: Post-procedure angiogram demonstrating restoration of distal arterial flow following angioplasty and stenting

In view of the chronic non-healing ulcer and diminished peripheral pulses, a cardiology consultation was sought. Arterial Doppler of the right lower limb demonstrated a mid-segment cut-off in the superficial femoral artery with reduced distal flow. Peripheral angiography performed on June 9, 2025, confirmed complete occlusion of the mid-superficial femoral artery with poor distal runoff, consistent with critical limb-threatening ischemia (Figure 2A). On June 10, 2025, the patient underwent percutaneous transluminal angioplasty with stenting of the right superficial femoral artery. The procedure was performed via an antegrade contralateral femoral approach, and the lesion was successfully crossed using a Command guidewire (Abbott Vascular Inc., Abbott Park, IL, USA). Pre-dilatation was carried out with a 5 × 80 mm balloon catheter, followed by deployment of a 5.5 × 200 mm Supera self-expanding stent (Abbott Vascular Inc.) across the diseased segment (Figure 2B). Final angiography demonstrated restoration of inline distal flow with achievement of Thrombolysis In Myocardial Infarction (TIMI) grade 3 flow and satisfactory distal runoff (Figure 2C). The procedure was completed without immediate complications. Post-procedurally, the patient received structured wound care, including regular sterile dressings, local debridement as required, pressure offloading of the affected heel, and optimization of glycemic control to facilitate ulcer healing.

Outcome and follow-up

**Figure 3:**
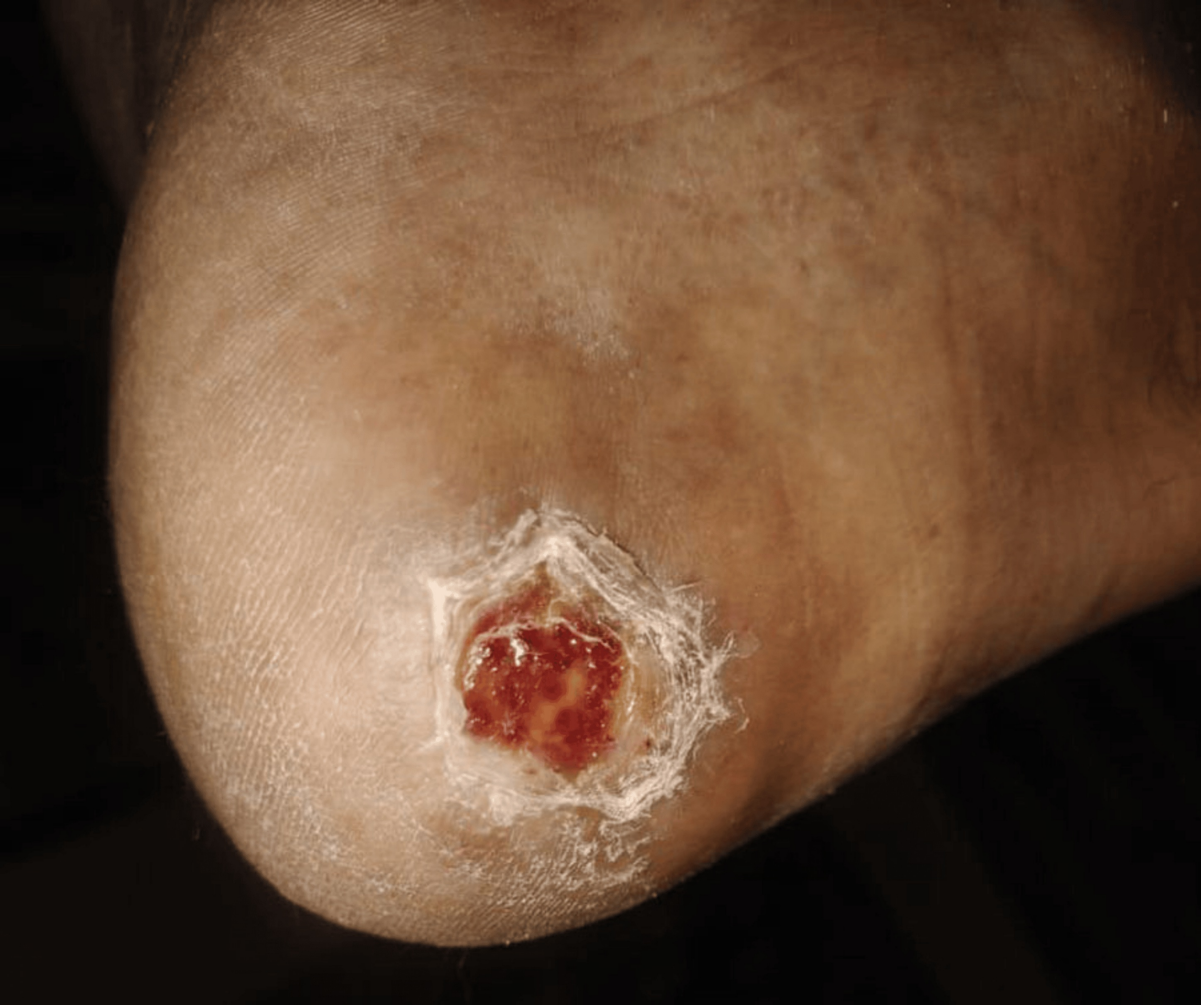
Early healing of the right heel ulcer with healthy granulation tissue following endovascular revascularization

**Figure 4:**
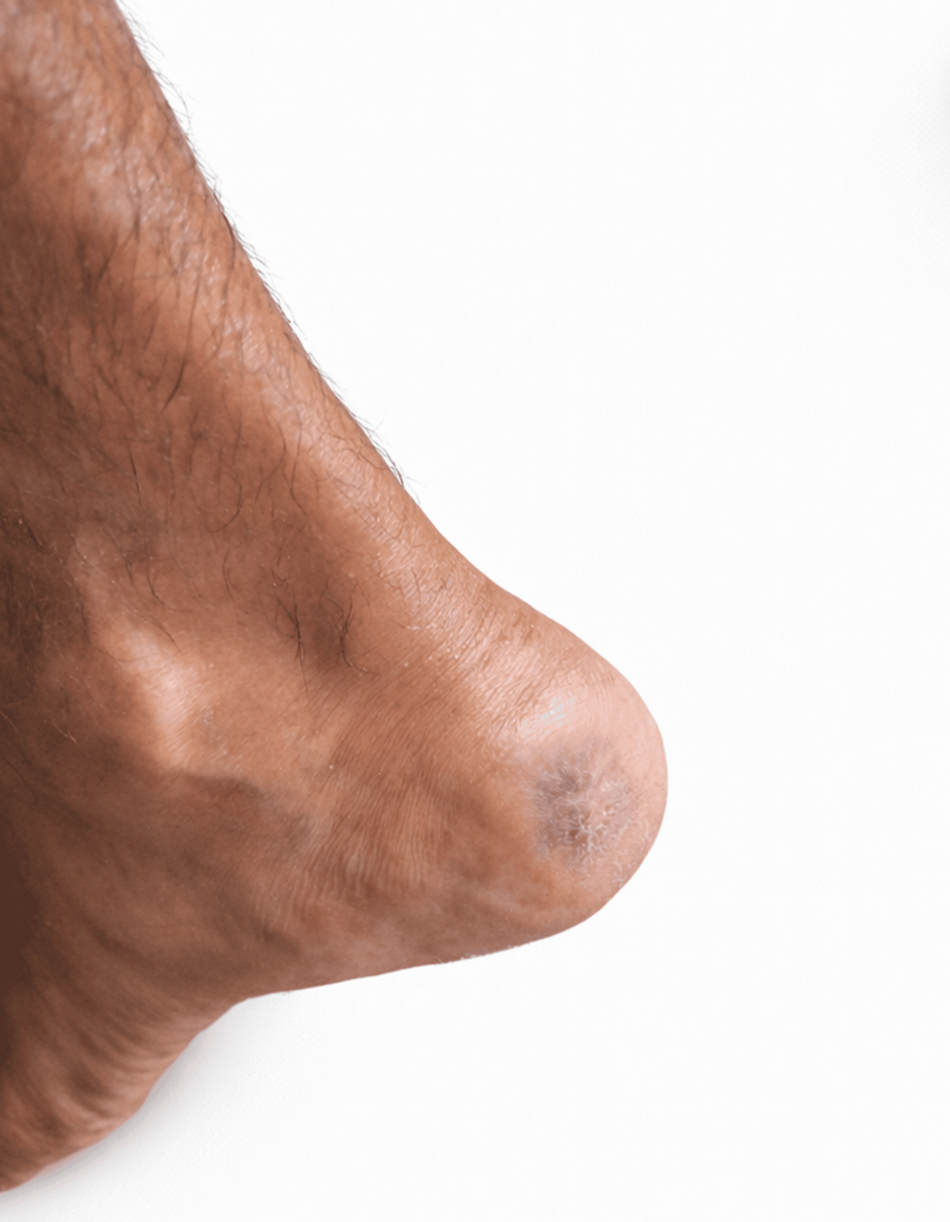
Complete epithelialization of the heel ulcer following endovascular revascularization

Following revascularization, the patient demonstrated progressive improvement in peripheral circulation, evidenced clinically by improved limb warmth and ulcer granulation. Over the subsequent weeks, the foot ulcer showed significant healing, as shown in Figure 3. Healing progressed steadily with healthy granulation and epithelialization and was achieved without the need for skin grafting or any reconstructive surgical intervention, indicating adequate tissue perfusion after successful endovascular revascularization. By August 6, 2025, the ulcer had completely healed with intact skin coverage (Figure 4), and the patient had regained full functional mobility and resumed his occupational duties without limitation. The patient remained clinically stable with no recurrence of ischemic symptoms during the available follow-up period.

## Discussion

Diabetic foot ulcers represent a complex clinical entity resulting from the interplay of peripheral neuropathy, vascular insufficiency, impaired immunity, and delayed wound healing. Among these factors, PAOD plays a decisive role in determining ulcer chronicity and the risk of progression to CLI, which is associated with a high probability of major amputation if not promptly managed.

In the present case, diabetes mellitus was diagnosed for the first time during evaluation for a chronic non-healing foot ulcer, highlighting the often silent nature of hyperglycemia until complications emerge. Similar observations have been reported by Armstrong et al., who emphasized that a significant proportion of diabetic foot ulcers serve as the initial clinical presentation leading to the diagnosis of diabetes or its complications, rather than glycemic symptoms themselves [4]. This underscores the importance of routine metabolic screening in patients presenting with chronic lower-limb wounds.

The presence of a non-healing ulcer for more than four months, along with diminished peripheral pulses, raised a strong suspicion of underlying vascular pathology in this patient. The International Working Group on the Diabetic Foot (IWGDF) guidelines stress that any diabetic foot ulcer failing to show signs of healing within four to six weeks should prompt evaluation for peripheral arterial disease [5]. In accordance with these recommendations, vascular imaging of our patient revealed PAOD with CLI, confirming that inadequate perfusion was a major contributor to delayed wound healing.

Several studies have demonstrated that PAOD significantly worsens outcomes in diabetic foot disease. Prompers et al., in a large European cohort, reported that ischemia was present in nearly half of patients with diabetic foot ulcers and was strongly associated with poor healing and increased amputation rates [6]. This finding closely parallels our case, where the ulcer remained refractory to medical therapy until the underlying ischemia was corrected.

Endovascular revascularization has emerged as a preferred strategy for limb salvage in selected patients with CLI, particularly those with infrainguinal disease. The TransAtlantic Inter-Society Consensus (TASC) II consensus and subsequent global vascular guidelines recommend revascularization, either surgical or endovascular, in all patients with CLI who are suitable candidates [7-8]. In the present case, percutaneous transluminal angioplasty with stenting of the superficial femoral artery successfully restored arterial flow, leading to rapid improvement in wound healing and limb viability.

In the present case, timely restoration of arterial inflow enabled complete ulcer healing without the need for skin grafting, highlighting the critical role of early endovascular intervention in limb salvage among patients with diabetic critical limb-threatening ischemia.

The effectiveness of revascularization in improving outcomes of diabetic foot ulcers has been well documented. Brouwer et al. demonstrated that correction of arterial insufficiency significantly improved ulcer healing rates and reduced the need for major amputations in diabetic patients with PAOD [9]. Similarly, a study by Elgzyri et al. concluded that revascularization markedly enhances limb salvage and ulcer healing in patients with diabetes and peripheral arterial disease, irrespective of the technique used [10]. The favorable outcome observed in our patient is consistent with these findings.

Another important aspect highlighted by this case is the value of a multidisciplinary approach. Management required coordination between internal medicine, diabetology, and cardiology to address metabolic derangements, renal dysfunction, and vascular pathology simultaneously. Conte et al., in the Global Vascular Guidelines, emphasized that integrated, team-based care is essential for optimal outcomes in patients with chronic limb-threatening ischemia, particularly those with diabetes [8].

Finally, this case reinforces that timely intervention can restore not only limb perfusion but also functional independence and quality of life. Delayed diagnosis of PAOD remains a major contributor to preventable amputations worldwide. Early recognition of vascular insufficiency and prompt endovascular intervention, as demonstrated in this case, can transform the prognosis of patients with diabetic foot ulcers.

## Conclusions

This case demonstrates that, in a patient with newly diagnosed diabetes and critical limb-threatening ischemia, early recognition of peripheral arterial disease and timely endovascular revascularization enabled complete ulcer healing and successful limb salvage without the need for skin grafting or reconstructive surgical intervention. Restoration of adequate arterial inflow facilitated progressive wound granulation and epithelialization, ultimately resulting in full functional recovery.

Although conclusions are limited by the single-case design and lack of extended long-term follow-up, this report underscores the importance of early vascular assessment in patients presenting with diabetic foot ulcers. Prompt identification and treatment of underlying arterial insufficiency may promote natural wound healing, reduce the need for additional surgical procedures, and decrease the risk of limb loss.
